# Pretreatment to terahertz absorption curves by narrow undulation constraint and Its quick implementation suggested by convex hull

**DOI:** 10.1038/s41598-022-21770-8

**Published:** 2022-10-24

**Authors:** Yizhang Li, Lingyu Liu, Ke Li, Zhongmin Wang, Tianying Chang, Wenqing Xu

**Affiliations:** Institute of Automation, Key Laboratory of UWB & THz of Shandong Academy of Sciences, Qilu University of Technology (Shandong Academy of Sciences), Jinan, China

**Keywords:** Applied mathematics, Computational science, Electrical and electronic engineering

## Abstract

In this work, a method of pretreating THz absorption curve is proposed, which leads to minimal range in absorption, reserving necessary undulation of curve for identification by convolutional neural network. The kernel thought of proposed method is about confining the undulation of curve with a pair of narrow parallel lines and solving their optimal position by consecutively rotation of normalized curve at two fixed points. A fast algorithm is further proposed based on features of convex hull, whose procedure is described in detail. The algorithm involves definition of some important point sets, calculating and comparing slopes and determining best choice out of 4 potential rotations. The rationality of searching critical point is illustrated in a geometric way. Additionally, the adaption of the method is discussed and real examples are given to show the capacity of method to extract nonlinear information of a curve. The study suggests that methods regarding computer graphics also contributes to feature extraction with respect to THz curve and pattern recognition.

## Introduction

Terahertz time domain spectroscopy is widely used for material detection and identification^1–4^. The curve of absorption or extinction coefficient is so related to the constituents of material that pattern recognition is conducted in various background^5–9^. Absorption peaks are not observed for pure substance with symmetrical molecular structure (for example, polyethylene). Besides, the peaks are less observable due to overlaps of component spectrum. Therefore, machine learning is significant for data mining in investigations involving but not limited to herbs, meats, tea, cereals according to previous reports. It is suggested that pretreatment to curves benefits model performance and reducing difficulty for training a satisfactory model. The conventional pretreatment includes Savitzky-Golay smoothing, filtering in frequency domain, multivariate scattering correction (MSC) and etc., which reserve essential feature for identification but adjust the value at every frequency sampling^10–12^. These algorithms assume the form of noise mathematically and all points are processed equally. However, the feature for identifying curves may be weakened and some parameter is empirically configured to obtain good results. In addition, methods involving computer graphics are seldom studied to bridge subsequent identification methods.

**Figure 1:**
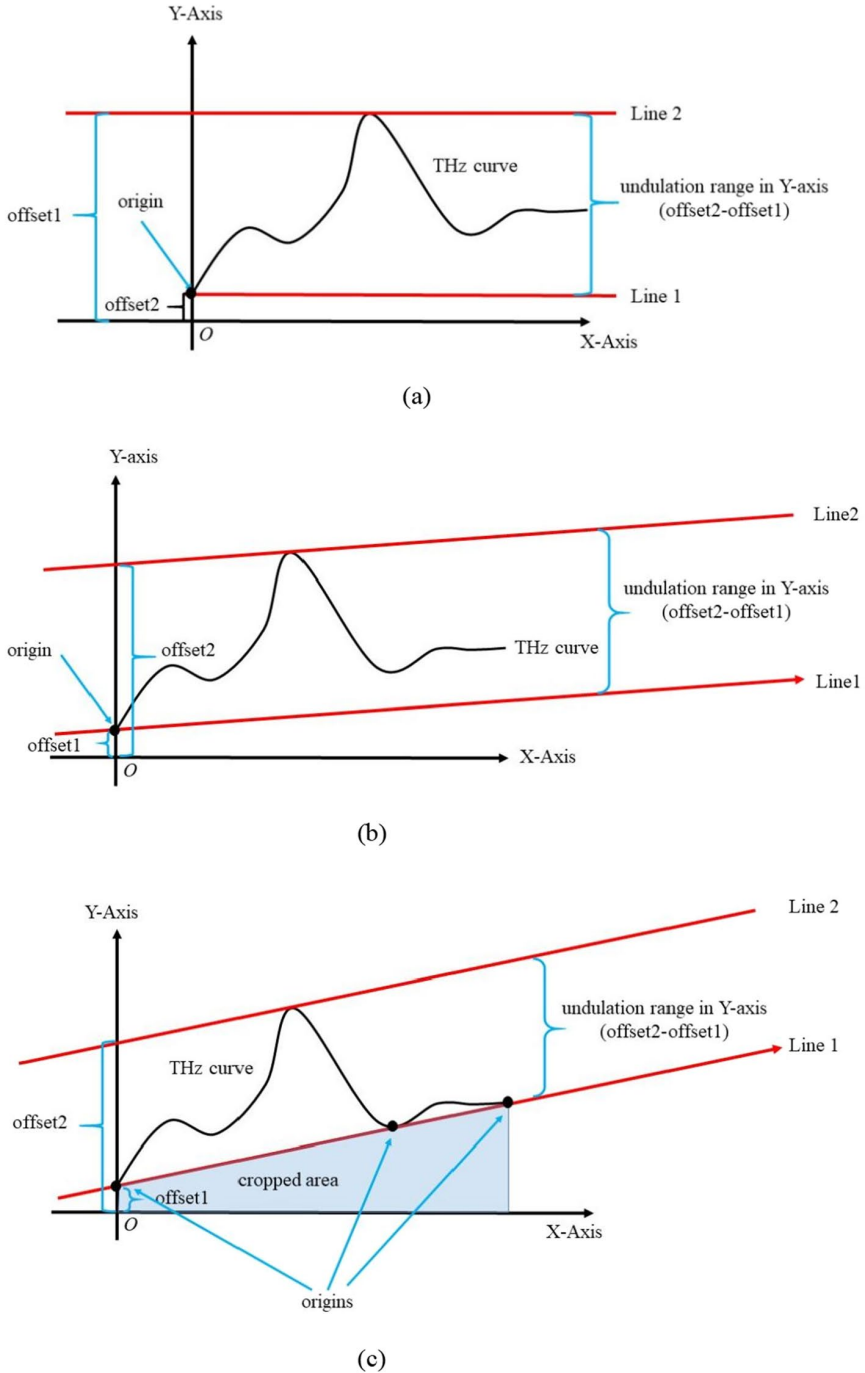
Schematic of Narrow Undulation Constraint: (**a**) original undulation in Y-axis; (**b**) adjusted undulation in Y-axis; (**c**) minimal undulation in Y-axis. Fig. 1 include 3 cases showing different undulation constraints. In Fig. 1a, the curve is confined by two lines parallel to X-axis (frequency axis). In this case, undulation in Y-axis direction equals the difference of maximum and minimum. In Fig. 1b, the curve is confined by two parallel lines and every line has one intersection point with curve. In this case, the undulation of curve in Y-axis direction can be further compressed if two parallel lines rotate around the present intersection points. In Fig. 1c, the curve is confined by two optimal parallel lines, of which one has one intersection point and the other has more than one intersection points with curve. In this case, the undulation of curve in Y-axis direction can’t be compressed any more. Apply shear transformation to narrow band indicated by two parallel lines, the difference in Y offset of parallel lines turn to new range in Y axis, which allows fewer pixels to quantify change in absorption.

Convolutional neural network (CNN) has been employed to identify object in an image as an effective and a popular model^13–16^. When CNN is associated with a terahertz curve, a conversion (or mapping) from THz curve to image is necessary before model training. The spectrum curve is viewed as a meaningful boundary in image to separate the upper part and lower part, which however, are meaningless because no actual value hits them. As a result, every pixel in an image participates in the training of CNN model. Compressing the range in absorption for given frequency band would meet the expectation of reducing computing cost, whereas the difficulty is to reserve essential features for identification. As is shown in Fig. 1a, a schematic THz curve has a range that equals the difference of offset1 and offset2. Line 2 and Line 1 are parallel lines with frequency axis, which indicate the upper and the lower bound in absorption. If another two parallel lines are used to confine curve, the undulation (difference in Y offset) between them changes (as is shown in Fig. 1b and Fig. 1c). Thus, we seek optimal parallel lines to confine curve with minimal distance and carry out shear transform to generate an image that accommodates the proceeded curve with minimal redundancy in Y direction (absorption dimension). Enlighted by the basic thought, the proposed method is named narrow undulation constraint (NUC).

In this study we propose an algorithm of NUC based on convex hull to fulfill optimal shear whose graphic annotation is provided to strengthen understanding of mathematic expression. It is believed that mathematic definitions are important for describing procedure of algorithm precisely but the rationality behind formulations is better accomplished via illustrations. It is suggested that methods regarding computer graphics is complementary to algebraic method in adjusting shape of curves, which may have an impact on THz spectrum analysis and facilitate applications of CNN models in THz curve identification.

## Method

### Formulation

Assuming we have a curve with *N* effective samplings (*N* ≥ 3) in frequency domain and the serial number of every point is determined by the ascending order of its frequency. The normalization is equivalent to a combination of translation and zooming. Then, the shear transformation is equivalent to cropping area beyond one of parallel lines by subtracting Y coordinates of one parallel lines from the normalized curve. Thus, we need matrices to denote input, translation, zoom factor, which are denoted by **X**_**N×2**_, **T**_**N×2**_, **Z**_**2×2**_ by (1), (2), (3) respectively where the subscript (or subscript in parenthesis) indicates the size of matrix. The final output of algorithm is denoted by Y in (4) and the range of its second column is expected to fall as much as possible, which is already described in introduction section. It is our goal to optimize **L**_N×2_, the matrix to denote cropping line.

1 $${\mathbf{X}} = \left[ {\begin{array}{*{20}c} {f_{1} } & {value\left( {f_{1} } \right)} \\ \vdots & \vdots \\ {f_{N} } & {value\left( {f_{N} } \right)} \\ \end{array} } \right]$$

2 $${\mathbf{T}} = \left[ {\begin{array}{*{20}c} { - f_{1} } & { - \min \left( {value} \right)} \\ \vdots & \vdots \\ { - f_{1} } & { - \min \left( {value} \right)} \\ \end{array} } \right]$$

3 $${\mathbf{Z}} = \left[ {\begin{array}{*{20}c} {\frac{1}{{f_{N} - f_{1} }}} & 0 \\ 0 & {\frac{1}{{max\left( {value} \right) - min\left( {value} \right)}}} \\ \end{array} } \right]$$

4 $${\mathbf{Y}} = \left( {\left( {{\mathbf{X}} + {\mathbf{T}}} \right){\mathbf{Z}} - {\mathbf{L}}} \right){\mathbf{Z}}^{ - 1} - {\mathbf{T}}$$

### Fast algorithm

It’s sensible to determine both the direction of cropping line and one point it goes through so as to have numeric expression of **L**. In other words, the optimization on L is equivalent to a task of point searching since the straight cropping line is determined by some critical points in the curve. Assume that the direction of cropping line is indicated by $\theta$, the acute angle between X-axis and cropping line. If the rotation from X-axis to cropping line is done counter clockwise, $\theta >0$; if the rotation is done clockwise, $\theta <0$. It is reasonable to conclude that $-0.5\pi <\theta <0.5\pi$ for all cases if cropping line are not orthogonal to X-axis.

**Figure 2:**
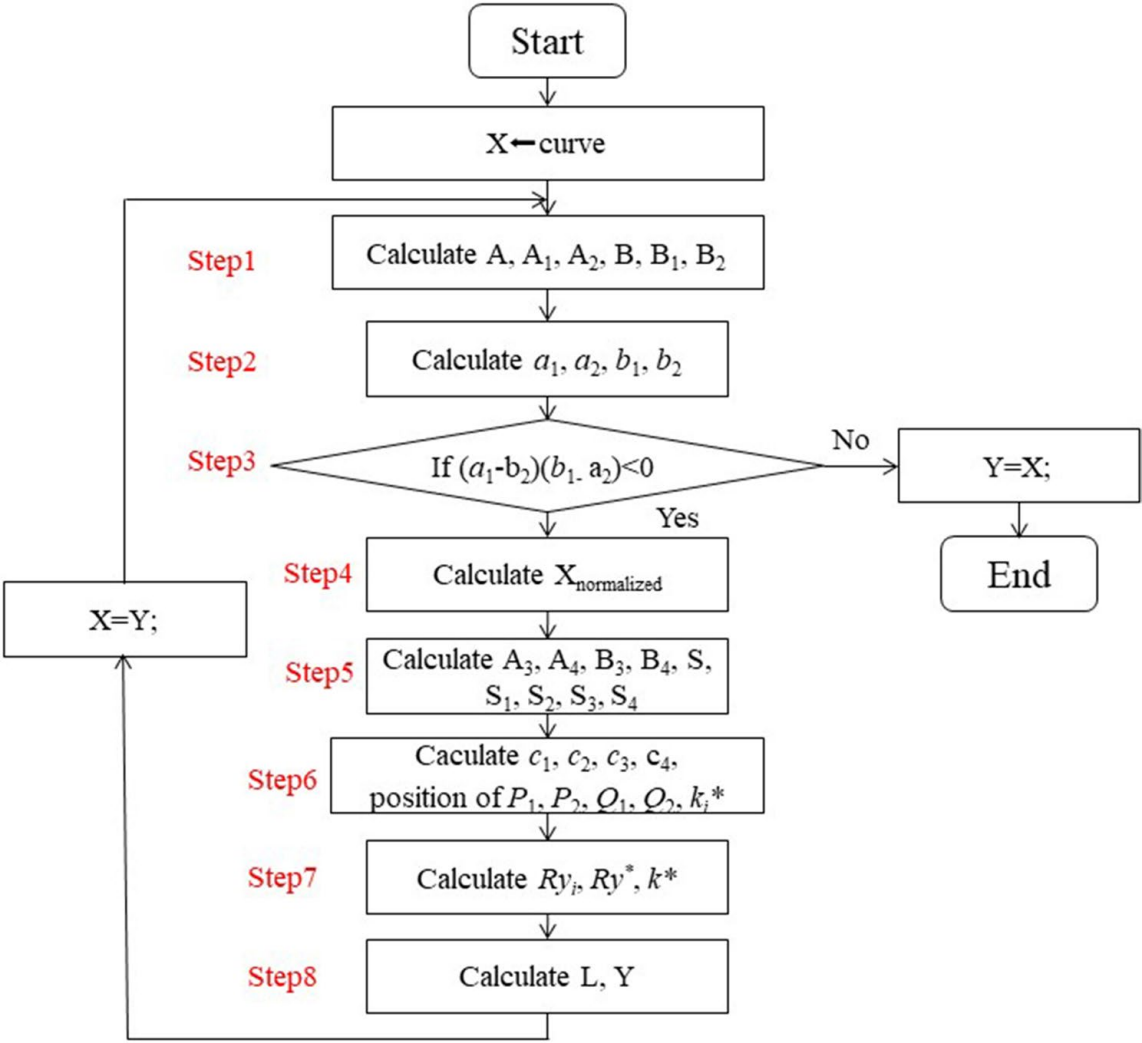
Schematic of the fast algorithm. Fig. 2 consists of all steps described in fast algorithm section. Note that there is an ‘if-else’ statement in block diagram, whose effect is to check the adaption of method to current curve (discussed in detail in Discussion Part).

The steps of algorithm are described in detail in this section and summarized in Fig. 2. Above all, the visualization of algorithm is discussed in next section.

*Step 1*: Organize potential critical points by sets.

The local minimum and local maximum of original curve at *f*_*i*_ are defined by (5) and (6) respectively. The bump point and the pit point at *f*_*i*_ are defined by (7) and (8) respectively. Build sets including A, A_1_, A_2_, B, B_1_ and B_2_ according to (9), (10), (11), (12), (13), (14) respectively. It is easy to prove that A_1_, A_2_, B_1_ and B_2_ are mutually exclusive; ${\mathrm{A}}_{1}\subset \mathrm{A}$ and ${\mathrm{B}}_{1}\subset \mathrm{B}$. For convenience, the element in set A and B are denoted by *a* and *b* respectively*.*

5 $$\left( {f_{i} ,{ }value(f_{i} } \right)){\text{ is a local minimum}},{\text{ s}}.{\text{t}}.{ }\left\{ {\begin{array}{*{20}c} {value\left( {f_{i} } \right) < value\left( {f_{i - 1} } \right)} \\ {value\left( {f_{i} } \right) < value\left( {f_{i + 1} } \right)} \\ \end{array} } \right.,{ }2 \le {\text{i}} \le N - 1$$

6 $$\left( {f_{i} ,{ }value(f_{i} } \right)){\text{ is a local minimum}},{\text{ s}}.{\text{t}}.{ }\left\{ {\begin{array}{*{20}c} {value\left( {f_{i} } \right) > value\left( {f_{i - 1} } \right)} \\ {value\left( {f_{i} } \right) > value\left( {f_{i + 1} } \right)} \\ \end{array} } \right.,{ }2 \le {\text{i}} \le N - 1$$

$$\left( {f_{i} ,{ }value(f_{i} } \right)){\text{ is a bump point}}$$

7 $${\text{ s}}.{\text{t}}.{ }\left\{ {\begin{array}{*{20}c} {\left( {value\left( {f_{i} } \right) - value\left( {f_{i - 1} } \right)} \right)\cdot\left( {value\left( {f_{i + 1} } \right) - value\left( {f_{i} } \right)} \right) > 0} \\ {value\left( {f_{i + 1} } \right) - value\left( {f_{i} } \right) < value\left( {f_{i} } \right) - value\left( {f_{i - 1} } \right)} \\ \end{array} } \right.$$

$$\left( {f_{i} ,{ }value(f_{i} } \right)){\text{ is a pit point}}$$,

8 $${\text{s}}.{\text{t}}. \left\{ {\begin{array}{*{20}c} {\left( {value\left( {f_{i} } \right) - value\left( {f_{i - 1} } \right)} \right)\cdot\left( {value\left( {f_{i + 1} } \right) - value\left( {f_{i} } \right)} \right) > 0} \\ {value\left( {f_{i + 1} } \right) - value\left( {f_{i} } \right) > value\left( {f_{i} } \right) - value\left( {f_{i - 1} } \right)} \\ \end{array} } \right.$$

9 $${\text{A}} = \{ i|value\left( {f_{i} } \right) \ge {\text{v}}alue\left( {f_{j} } \right),{ }1 \le i,{ }j \le N,{ }i{\text{ and }}j{\text{ are integers}}\}$$

10 $${\text{A}}_{1} = \{ i{|} \left( {f_{i} ,value\left( {f_{i} } \right)} \right) {\text{is a local maximum}}\}$$

11 $${\text{A}}_{2} = \{ i{|} \left( {f_{i} ,value\left( {f_{i} } \right)} \right) {\text{is a bump}}\}$$

12 $${\text{B}} = \{ i|value\left( {f_{i} } \right) \le {\text{v}}alue\left( {f_{j} } \right),{ }1 \le i,{ }j \le N,{ }i{\text{ and }}j{\text{ are integers}}\}$$

13 $${\text{B}}_{1} = \{ i{|} \left( {f_{i} ,value\left( {f_{i} } \right)} \right) {\text{is a local minimum}}\}$$

14 $${\text{B}}_{2} = \{ i{|} \left( {f_{i} ,value\left( {f_{i} } \right)} \right) {\text{is a pit}}\}$$

*Step 2*: Record indexes for potential rotation centers based on Step 1.

The calculation of *a*_1_, *a*_2_, *b*_1_ and *b*_2_ is shown in (15), (16), (17), (18) respectively. Particularly, the expression ‘min’ denotes the operation to get minimal value in the set while the expression ‘max’ denotes the operation to get maximal value in the set. *P*_1_ and *P*_2_ are both global maximum of the curve; *Q*_1_ and *Q*_1_ are both global minimum of the curve. In particular, *P*_1_ and *P*_2_ overlaps if Card(A_1_) = 1, thus *a*_1_ = *a*_2_; *Q*_1_ and *Q*_2_ overlaps if Card(B_1_) = 1, thus *b*_1_ = *b*_2_. The expression ‘Card’ means to obtain total number of elements in the set (cardinal number).

15 $$a_{1} = {\text{min}}\left( {\text{A}} \right)$$

16 $$a_{2} = {\text{max}}\left( {\text{A}} \right)$$

17 $$b_{1} = {\text{min}}\left( {\text{B}} \right)$$

18 $$b_{2} = {\text{max}}\left( {\text{B}} \right)$$

*Step 3*: Check if curve can be processed effectively by the algorithm.

Check if (*a*_1_−*b*_2_)(*b*_1_−*a*_2_) < 0. If this condition is met, the curve can be processed effectively. Just follow Step 4–8 and **Y** = **X**, then restart from Step 1. If (*a*_1_-*b*_2_)(*b*_1_-*a*_2_) > 0, the current **X** would be final output.

*Step 4*: Normalize original curve and obtain coordinate in Euclidean Space.

The position of every point in Euclidean Space after normalization can be determined by (19) where the definition of matrix Z is found in (3). The coordinate of normalized points is a function of serial number indicated by (20) and (21). After normalization, the curve is inlaid into a square area defined by {(0,0), (1,0), (1,1), (0,1)} in Euclidean Space whose edge equals one. The normalized curve has at least one intersection point with every edge. It is important that the type of one point does not change after normalization because the normalization have no impact on the sorting of values. For example, one point is known as a bump point in original coordinate and it’s still a bump point after normalization although the coordinate values for two directions change. Such phenomena can be generalized to points marked as pit, local minimum, local maximum.

19 $${\mathbf{X}}_{{{\mathbf{normalized}}}} = \left( {{\mathbf{X}} + {\mathbf{T}}} \right){\mathbf{Z}}$$

20 $$x\left( i \right) = \frac{{f_{i} - f_{1} }}{{f_{N} - f_{1} }}{ }1 \le i \le N, i{\text{ is an integer}}$$

21 $$y\left( i \right) = \frac{{value(f_{i} ) - value(f_{b} )}}{{value(f_{a} ) - value(f_{b} )}}{ }1 \le i \le N, i{\text{ is an integer}},{ }a \in {\text{A}},{ }b \in {\text{B}}$$

Obviously, according to our definition, we get *y*(*a*_1_) = *y*(*a*_2_) = 1 and *y*(*b*_1_) = *y*(*b*_2_) = 0.

*Step 5*: Calculate slope of probable edge of convex hull in Cartesian coordinate.

Four sets to accommodate potential edge point of convex hull are built according to (22), (23), (24) and (25), respectively. Set S, the union of 4 mutually exclusive subsets S_1_, S_2_, S_3_ and S_4_ (indicated by (26)), contains all the slope values for comparison. The specific definition of S_1_, S_2_, S_3_ and S_4_ is shown in (27), (28), (29) and (30) respectively. The slope between two points whose indexes are *i* and *j* is calculated according to (31) where 1 ≤ *i*, *j* ≤ *N*, *i* and *j* are integers.

22 $${\text{A}}_{3} = \left\{ 1 \right\} \cup \left\{ {i{|}i \in ({\text{A}}_{1} \cup {\text{A}}_{2} ), i < a_{1} } \right\}$$

23 $${\text{A}}_{4} = \left\{ N \right\} \cup \left\{ {i{|}i \in \left( {{\text{A}}_{1} \cup {\text{A}}_{2} } \right), i > a_{2} } \right\}$$

24 $${\text{B}}_{3} = \left\{ 1 \right\} \cup \left\{ {i{|}i \in \left( {{\text{B}}_{1} \cup B_{2} } \right), i < b_{1} } \right\}$$

25 $${\text{B}}_{4} = \left\{ N \right\} \cup \left\{ {i{|}i \in \left( {{\text{B}}_{1} \cup B_{2} } \right), i > b_{2} } \right\}$$

26 $${\text{S}} = {\text{S}}_{1} \cup {\text{S}}_{2} \cup {\text{S}}_{3} \cup {\text{S}}_{4}$$

27 $${\text{S}}_{1} = \{ {\text{s}}\left| {{\text{s}} = } \right|slope\left( {i,a_{1} } \right)|,i \in {\text{A}}_{3} \}$$

28 $${\text{S}}_{2} = \{ {\text{s}}\left| {{\text{s}} = } \right|slope\left( {i,a_{2} } \right)|,i \in {\text{A}}_{4} \}$$

29 $${\text{S}}_{3} = \{ {\text{s}}\left| {{\text{s}} = } \right|slope\left( {i,b_{1} } \right)|,i \in {\text{B}}_{3} \}$$

30 $${\text{S}}_{4} = \{ {\text{s}}\left| {{\text{s}} = } \right|slope\left( {i,b_{2} } \right)|,i \in {\text{B}}_{4} \}$$

31 $$slope\left( {i,j} \right) = \left\{ {\begin{array}{*{20}c} {\frac{y\left( i \right) - y\left( j \right)}{{x\left( i \right) - x\left( j \right)}}, i \ne j} \\ {0, i = j} \\ \end{array} } \right.$$

*Step 6*: Mark potential rotation and critical points for distance check.

Serial number *c*_1_, *c*_2_, *c*_3_ and *c*_4_ are used to indicate points in the convex hull of the normalized curve, which play a role in further calculations. The stipulation of them are seen in (32), (33), (34) and (35) respectively.

32 $$c_{1} = {\text{min}}\left( {{\text{C}}_{1} } \right), {\text{s}}.{\text{t}}. {\text{C}}_{1} = \left\{ {i{|}i \in {\text{A}}_{3} , \left| {slope\left( {i,a_{1} } \right)} \right| = \min \left( {{\text{S}}_{1} } \right)} \right\}$$

33 $$c_{2} = {\text{max}}\left( {{\text{C}}_{2} } \right), {\text{s}}.{\text{t}}. {\text{C}}_{2} = \left\{ {i{|}i \in {\text{A}}_{4} , \left| {slope\left( {i,a_{2} } \right)} \right| = \min \left( {{\text{S}}_{2} } \right)} \right\}$$

34 $$c_{3} = {\text{min}}\left( {{\text{C}}_{3} } \right), {\text{s}}.{\text{t}}. {\text{C}}_{3} = \left\{ {i{|}i \in {\text{B}}_{3} , \left| {slope\left( {i,b_{1} } \right)} \right| = \min \left( {{\text{S}}_{3} } \right)} \right\}$$

35 $$c_{4} = {\text{max}}\left( {{\text{C}}_{4} } \right), {\text{s}}.{\text{t}}. {\text{C}}_{4} = \left\{ {i{|}i \in {\text{B}}_{4} , \left| {slope\left( {i,b_{2} } \right)} \right| = \min \left( {{\text{S}}_{4} } \right)} \right\}$$

The coordinate of *P*_1_, *P*_2_, *Q*_1_, *Q*_2_ are confirmed depending on the rotation angle *θ* and rotation center. Assume parallel lines are named line1 and line 2 according to Y offset (offset2 > offset1). A universal rule to claim *P*_1_, *P*_2_, *Q*_1_, *Q*_2_ is described as follow:

*P*_i_ denotes point (the fixed rotation center or the critical point to be found) in line 1 and *Q*_i_ denotes point (the fixed rotation center or the critical point to be found) in line 2. If critical edge of convex hull is determined by line 1 (in case 1 and case 2), *Q*_1_ and *Q*_2_ overlaps. If critical edge of convex hull is supposed to betermined by line 2 (in case 3 and case 4), *P*_1_ and *P*_2_ overlaps. (*x*(*a*_1_), *y*(*a*_1_)) and (*x*(*b*_2_), *y*(*b*_2_)) are fixed as rotation center of line 2 and line 1, respectively in both case 1 and case 4. As a contrast, (*x*(*a*_2_), *y*(*a*_2_)) and (*x*(*b*_1_), *y*(*b*_1_)) are fixed as rotation center of line 2 and line 1, respectively in both case 2 and case 3. *P*_1_ lies to the left of *P*_2_ unless they overlap; *Q*_1_ lies to the left of *Q*_2_ unless they overlap.

A deduction of above-mentioned rule in 4 cases are described as follow:

#### Case 1

If *point* (*x*(*a*_1_), *y*(*a*_1_)) is fixed as rotation center, *P*_1_ is defined as *P*_1_(*x*(*c*_1_), *y*(*c*_1_)) and *P*_2_ is defined as *P*_2_(*x*(*a*_1_), *y*(*a*_1_)). Meanwhile, *Q*_2_ is defined as *Q*_2_(*x*(*b*_2_), *y*(*b*_2_)) and *Q*_1_ overlaps with *Q*_2_.

#### Case 2

If point (*x*(*a*_2_), *y*(*a*_2_)) is *fixed* as rotation center, *P*_1_ is defined as *P*_1_(*x*(*a*_2_), *y*(*a*_2_)) and *P*_2_ is defined as *P*_2_(*x*(*c*_2_), *y*(*c*_2_)). Meanwhile, *Q*_1_ is defined as *Q*_1_(*x*(*b*_1_), *y*(*b*_1_)) and *Q*_2_ overlaps with *Q*_1_.

#### Case 3

If point (*x*(*b*_1_), *y*(*b*_1_)) is fixed as rotation center, *P*_2_ is defined as *P*_2_(*x*(*a*_2_), *y*(*a*_2_)) and *P*_1_ overlaps with *P*_2_. *Meanwhile*, *Q*_1_ is defined as *Q*_1_(*x*(*c*_3_), *y*(*c*_3_)) and *Q*_2_ is defined as *Q*_2_(*x*(*b*_1_), *y*(*b*_1_)).

#### Case 4

If point (*x*(*b*_2_), *y*(*b*_2_)) is fixed as *rotation* center, *P*_1_ is defined as *P*_1_(*x*(*a*_1_), *y*(*a*_1_)) and *P*_2_ overlaps with *P*_1_. Meanwhile, *Q*_1_ is defined as *Q*_1_(*x*(*b*_2_), *y*(*b*_2_)) and *Q*_2_ is defined as *Q*_2_(*x*(*c*_4_), *y*(*c*_4_)).

Four possible rotation angles expressed by its tan function is found in (36).

36 $$k_{i}^{*} = \left\{ {\begin{array}{*{20}c} {\begin{array}{*{20}c} {slope\left( {c_{1} ,a_{1} } \right), i = 1} \\ {slope\left( {c_{2} ,a_{2} } \right), i = 2} \\ \end{array} } \\ {slope\left( {c_{3} ,b_{1} } \right), i = 3} \\ {slope\left( {c_{4} ,b_{2} } \right), i = 4} \\ \end{array} } \right.$$

*Step 7*: Determine optimal rotation on account of slopes and distance in Y direction.

Assume that the convex hull of curve will be confined by a pair of parallel lines that penetrate at least three points in its edge. The line with minor offset is named Line 1 and the other is named Line 2. The difference between offset of Line 2 and Line 1 is formulated by (37). The optimal Ry* is found in (38). Correspondingly, k* is determined according to (39). After comparing *Ry*_*i*_, only one case is reserved as the final result and the corresponding rotation centers are recorded.

37 $$Ry_{i} = \left\{ {\begin{array}{*{20}c} {1 + (b_{2} - a_{1} )*min\{ |k_{1}^{*} \left| , \right|k_{4}^{*} |\} , i = 1or 4} \\ {1 + \left( {a_{2} - b_{1} } \right)*min\left\{ {\left| {k_{2}^{*} } \right|,\left| {k_{3}^{*} } \right|} \right\}, i = 2 or 3} \\ \end{array} } \right.$$

38 $$Ry^{*} = {\text{min}}\left\{ {Ry_{1} , Ry_{2} ,Ry_{3} , Ry_{4} } \right\}$$

39 $$k^{*} = k_{i}^{*} {\text{s}}.{\text{t}}.\left\{ {\begin{array}{*{20}c} {Ry^{*} = Ry_{i} ,Ry^{*} = Ry_{j} } \\ {\left| {k_{i}^{*} \left| \le \right|k_{j}^{*} } \right|} \\ \end{array} } \right. , i,j \in \left\{ {1,2,3,4} \right\}, i < j$$

*Step 8*: Adjust curve in original space according to previous calculation.

Line 1 is denoted by **L** in matrix. The offset of Line 1 in Y axis is calculated by (40). The equation of Line for the investigated internal is formulated according to (41). After obtaining **L**, we get the adjusted curve according to (4).

40 $$offset_{1} = \left\{ {\begin{array}{*{20}c} { - b_{2} k^{*} , k^{*} = k_{1}^{*} } \\ { - b_{1} k^{*} , k^{*} = k_{2}^{*} } \\ { - b_{1} k^{*} , k^{*} = k_{3}^{*} } \\ { - b_{2} k^{*} , k^{*} = k_{4}^{*} } \\ \end{array} } \right.$$

41 $${\mathbf{L}} = \left[ {\begin{array}{*{20}c} {\begin{array}{*{20}c} 0 & {k^{*} x_{1} + offset_{1} } \\ \vdots & \vdots \\ \end{array} } \\ {\begin{array}{*{20}c} 0 & {k^{*} x_{N} + offset_{1} } \\ \end{array} } \\ \end{array} } \right]$$

### Geometric Interpretation

Given that the meaning of matrices, points and sets is abstract, we show how the algorithm is designed with help of figures. It’s necessary to claim that the ‘curve’ shall be understood as ‘polylines’ because of sampling and that a true curve does not exist in digital system. The polylines look as smooth as a curve if the change is not drastic in scale of sampling.

**Figure 3:**
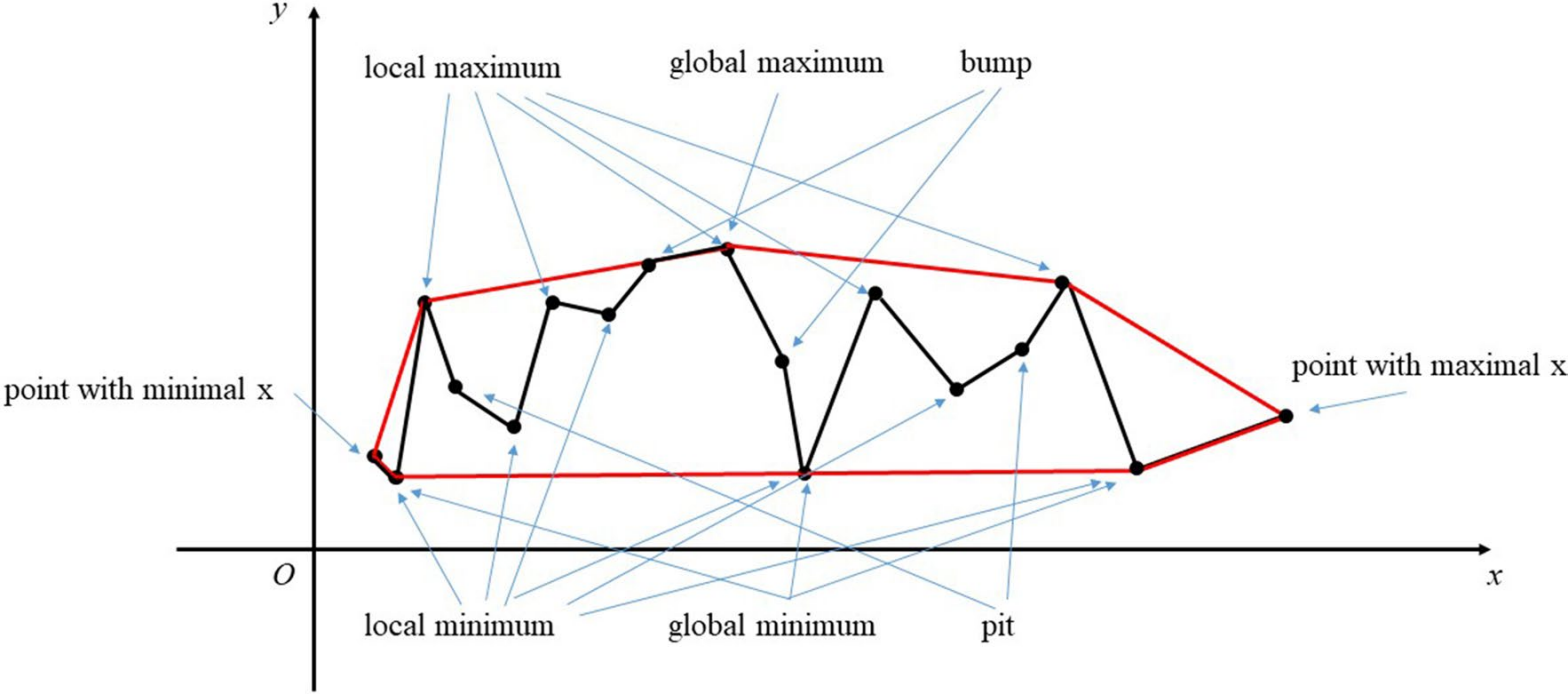
Schematic of convex hull and critical points defined in this paper. Fig. 3 gives examples of bump, pit, local maximum, local minimum, global maximum, global minimum. Point with maximal x and minimal x (x can be replaced by serial number) set the right and left boundary of curve, respectively. In addition, points with consecutive serial number but same y value do not belong to any above-mentioned point set, which are not depicted in this figure. They are not employed in this algorithm and do not affect result either.

**Figure 4:**
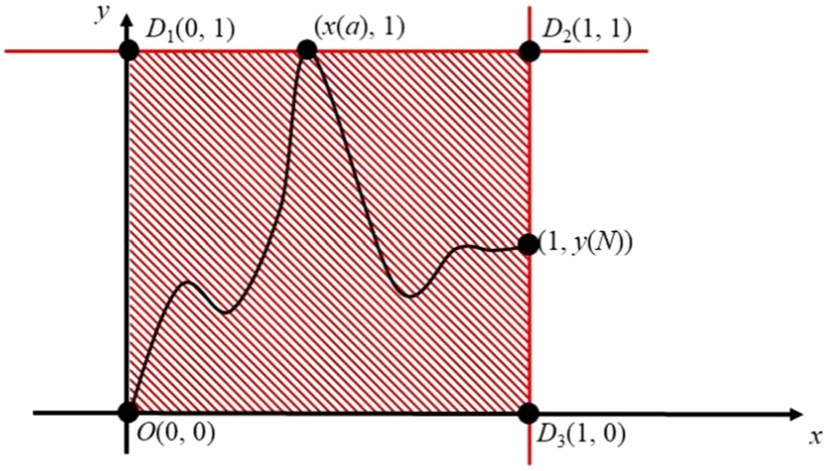
Schematic of normalized curve in a square. In Fig. 4, the normalized curve is laid in a square which is defined by {(0,0), (0,1), (1,1), (1,0)}. The curve has one intersection point with OD_1_ and D_2_D_3_ respectively regardless of curve shape although the intersection point can be any of O(0,0), D1(0,1), D_2_(1,1) and D_3_(1,0). However, with the change of curve, one or more intersection points are allowed to find in D_1_D_2_ and OD_3_.

Assume there is a set of points S, and convex hull is the intersection of all convex sets containing S^17–19^, which has been widely used in computer graphics. In a plane, a convex hull is the polygon with minimal area to cover all points. Some widely used convex hull algorithms are gift wrapping^20^, Graham scan^21^, quick hull^22^, divide and conquer^23^ and monotone chain^24^. A schematic of convex hull is presented in Fig. 3 that has 7 segments as edges in red. The example curve is connected by 16 segments in black and three of them overlaps with the edge of convex hull. According to local variation of curve, we build point set A, A_1_, A_2_, B, B_1_, B_2_ according to expressions in (9)–(14). The representative points are marked using blue arrows. The direction change between two adjacent edges of convex hull is not allowed to increase further for given set S. As a result, gift wrapping algorithm is designed to obtain convex hull. In Andrew’s Algorithm^24^, the search of convex hull starts from point with extreme X or Y value, we develop this thought to process normalized curve, where both dimension difference and range difference in X and Y direction are removed. In Fig. 4, the normalized curve has intersections with segment *D*_1_*D*_2_ and *OD*_3_ that indicate the two parallel lines to confine undulation presently. In addition, the convex hull of polyline is a polygon that now locates in the square OD_1_D_2_D_3_. In our work, we make use of convex hull to find optimal adjustment of curves by searching critical edges, however, we do not need to obtain entire convex hull. As a result, the proposed method is aimed at calculate convex hull.

**Figure 5:**
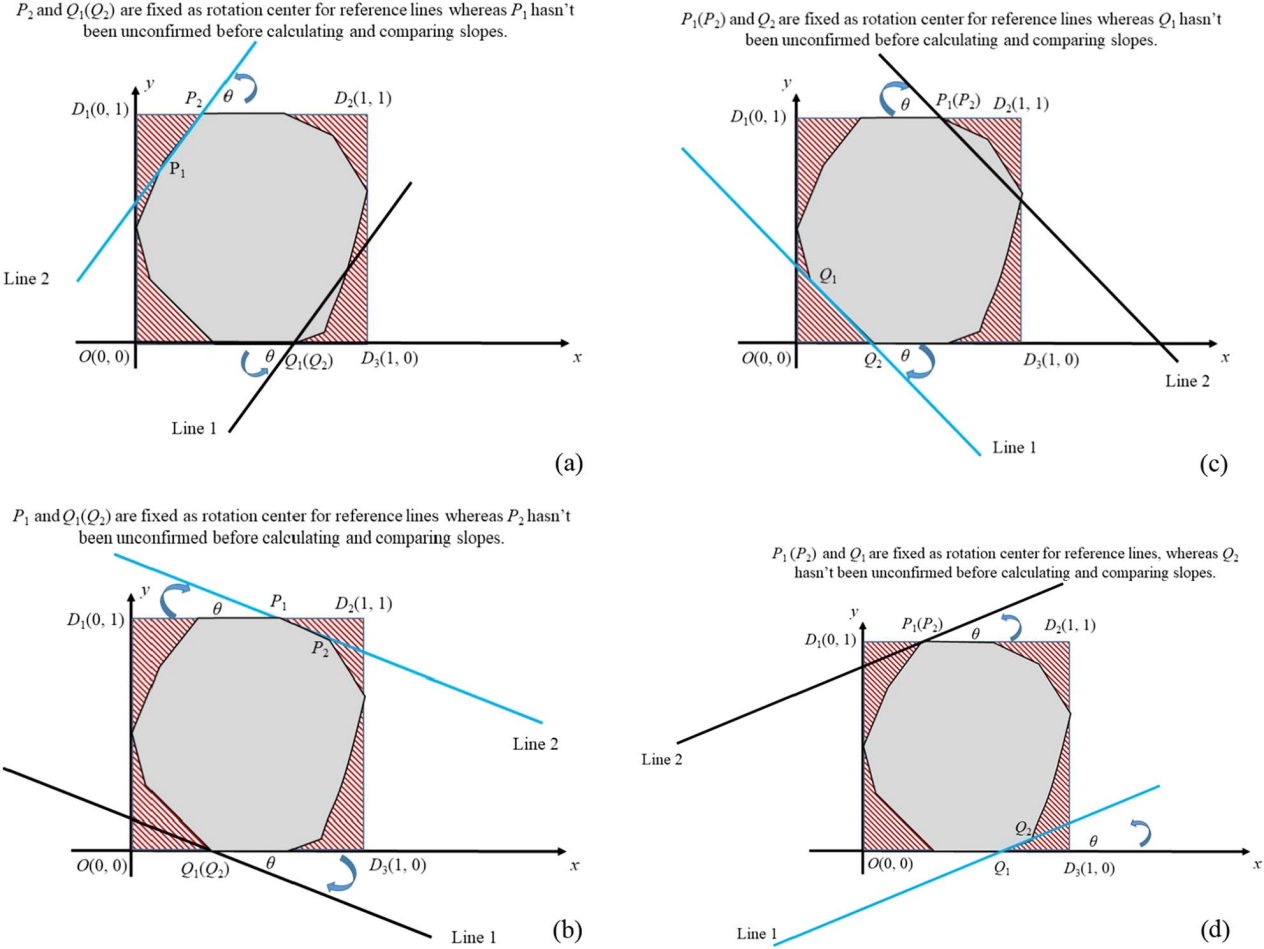
Schematic of searching critical edge of convex hull. Fig. 5 involves 4 cases to search critical edge of convex hull, which starts by rotating line that go through the point in northwest, northeast, southwest, southeast as the rotation center, respectively. The gray polygon indicates the area that a typical convex hull covers. Imagine *θ* changes continuously from 0 for both parallel lines and if |*θ|* is big enough, a region formed by one of parallel lines and part of convex hull is found, proving that at least one vertice of convex hull lie beyond the narrow band. As the processed undulation change monotonically with *θ*, we just calculate extreme cases and select the optimal one out of four. One critical point to determine parallel lines belong to set A_3_ A_4_ B_3_ B_4_ respectively in 4 cases, respectively. The other critical point is (*x*(*a*_1_),1), (*x*(*a*_2_),1) (*x*(*b*_2_),0) and (*x*(*b*_1_),0) for 4 cases.

As is mentioned, two parallel lines for reference are employed to confine undulation and one of them would coincide with one edge of convex hull since the continuous rotation of reference line centered at some point is like wrapping in Andrew’s Algorithm. In our algorithm, the parallel line determined by convex hull is described as ‘dominating parallel line’ and the other as ‘following parallel line’. The difference in offset of two parallel lines is the undulation we try to confine. Above all, to ensure wrapping is valid, we start from points with extreme Y values including (*x*(*a*_1_),1), (*x*(*a*_2_),1), (*x*(*b*_2_),0) and (*x*(*b*_1_),0) as fixed rotation center. The details are illustrated in Fig. 5 in 4 cases that correspond with the descriptions in ‘Fast Algorithm’ part.

**Figure 6:**
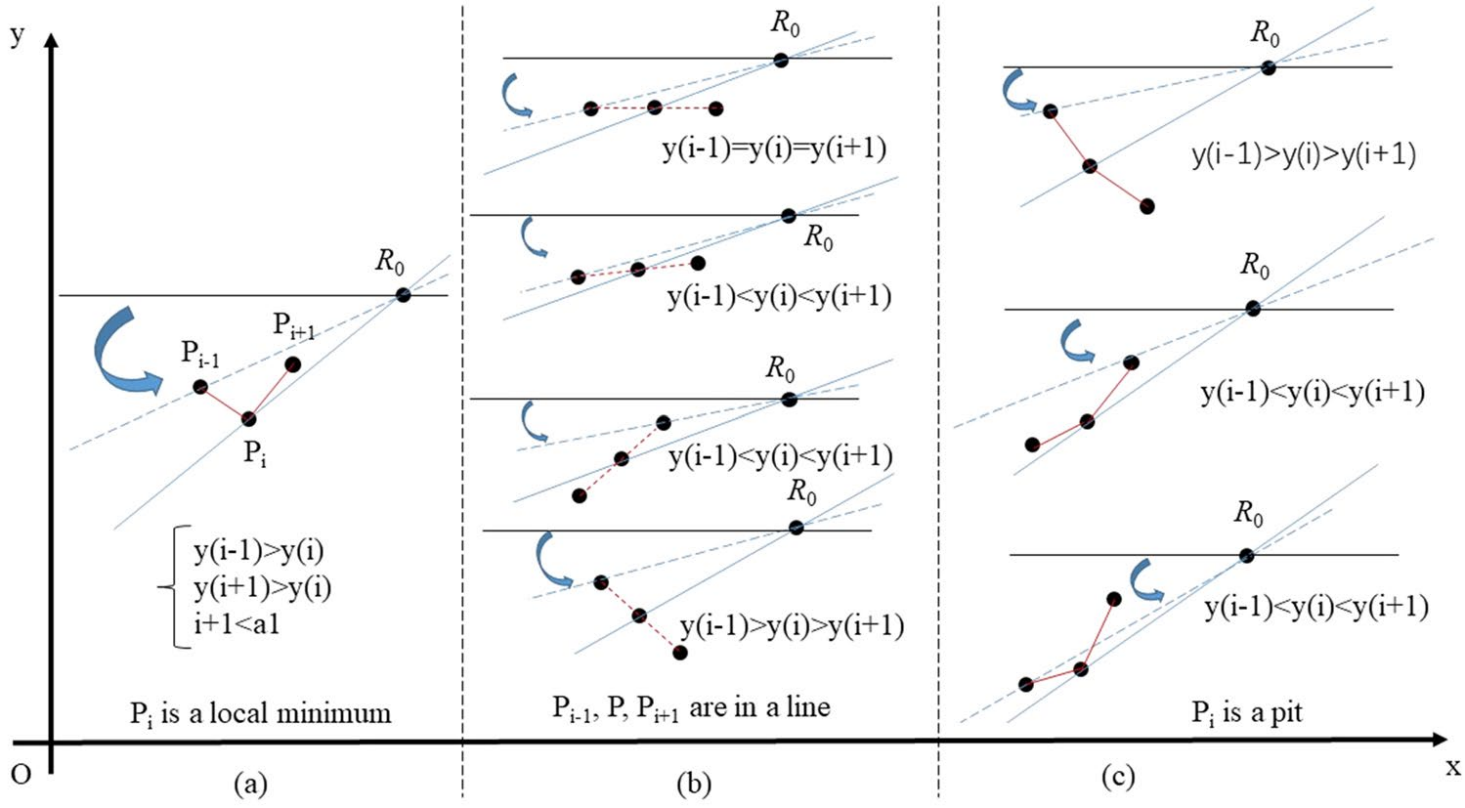
Rejection subcases in searching critical points: rotating line with 0 < *θ* < 0.5π in case 1 and case 4: (**a**) any local minimum (**b**) any internal point aligned in curve (**c**) any pit. Fig. 6 shows why some points are ignored during calculating slopes. According to Fig. 6, with the increasing of *θ*, solid black line would coincide with blue dash line first and with blue solid line then, indicating P_i_ loses its qualification as a vertice in convex hull of curve. As a special case where red dash line coincides with blue dash line (not depicted), P_i-1_, P_i_, P_i+1_ would contribute to the same *k*_1_^*^ and P_i_ is also not a vertice in convex hull.

**Figure 7:**
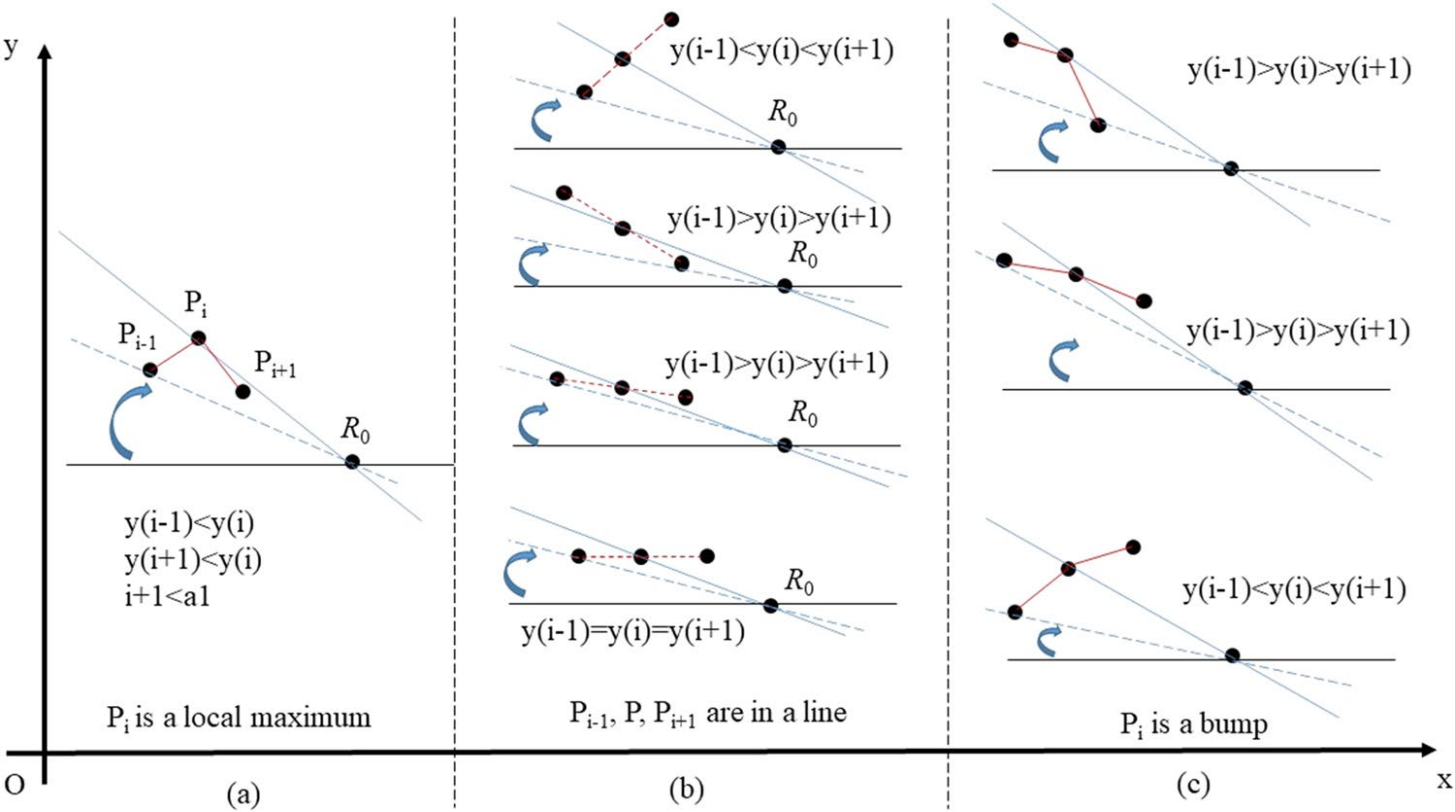
Rejection subcases in searching critical points: rotating line with −0.5π < *θ* < 0 in case 2 and case 3: (**a**) any local maximum (**b**) any internal point aligned in curve (**c**) any bump. According to Fig. 7, with the decreasing of *θ*, solid black line would coincide with blue dash line first and with blue solid line then, indicating P_i_ loses its qualification as a vertice in convex hull of curve. As a special case where red dash line coincides with blue dash line (not depicted), P_i-1_, P_i_, P_i+1_ would contribute to the same *k*_3_^*^ and P_i_ is also not a vertice in convex hull.

In Fig. 5, the area of convex hull is depicted using a polygon in gray and the dominating parallel line is highlighted in blue. As the rotation direction is limited in 4 cases, we search another point to determine dominating parallel line by calculating the slope of line that penetrates (*x*(*a*_1_),1), (*x*(*a*_2_),1), (*x*(*b*_2_),0) and (*x*(*b*_1_),0), respectively. Due to the particularity of location of the fixed rotation centers, only a part of points is utilized, which reduces the calculation cost. The rejection subcases with respect to case 1 and case 3 are illustrated in Fig. 6, and the slope calculation is omitted for the points mentioned. Therefore, we conduct set operations in Eq. (22) and Eq. (24). Similarly, some points can also be filtered before calculating slope in case 2 and case 4, which corresponds with regulations in Eq. (23) and Eq. (25). As an exception, the slope of lines parallel with Y axis are defined as 0, which does not conform to corresponding mathematic term. Once the cardinal number of A_3_, A_4_, B_3_, B_4_ reaches 1, the index ‘*slope*’ would be a number defined by one point itself. The treatment in Eq. (31) is to ensure robustness of algorithm.(Fig. 7).

**Figure 8:**
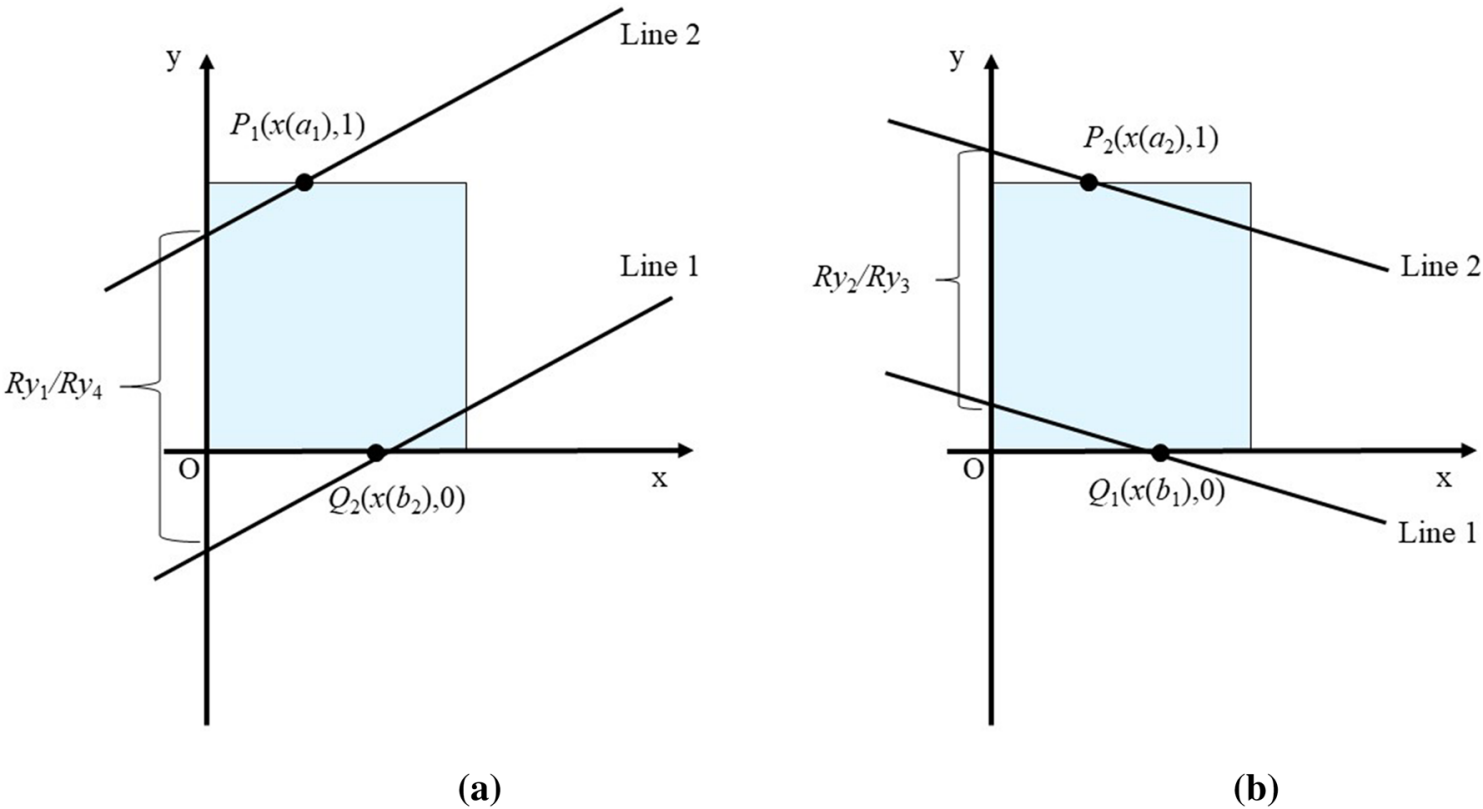
Calculation of distance between parallel lines in Y-axis: (**a**) *Ry*_1_ or *Ry*_4_ (**b**) *Ry*_2_ or *Ry*_3_. The calculation of *Ry*_*i*_ in Case 1 and case 4 (i.e., *i* = 1 or 4) is shown in Fig. 8a ; the calculation of *Ry*_*i*_ in Case 2 and case 3 (i.e., *i* = 2 or 3) is shown in Fig. 8b. It is easy to get (37) with elementary geometry and trigonometric function.

However, confine all points within band determined by convex hull is not always successful if only one parallel line is considered (in Fig. 5a and Fig. 5c, at least one vertice of convex hull lies beyond the band). Thus, we need to select the appropriate rotation (indicated by k*) from candidate ones (*k*_i_*). The undulation in Y-axis between parallel lines (offset difference in Y-axis, denoted by *Ry*_*i*_) is derived according to Fig. 8. Note that the difference between *Ry*_*i*_ and 1 is proportional to the absolute tan of angle. Thus, the four *Ry*_*i*_ indicates the utmost allowed rotation. Calculate Ry* and k* according to (38), (39) before deriving L based on slope and one point it goes through. In case 1 and case 4, the rotation center of line with minor offset (cropping line, line 1) in Y-axis is marked *Q*_1_; in case 2 and case 3, the rotation center of cropping line in Y-axis is marked *Q*_2_. After obtaining **L**, the curve is normalized reversely according to (4) and the range of undulation is reduced.

## Results

**Figure 9:**
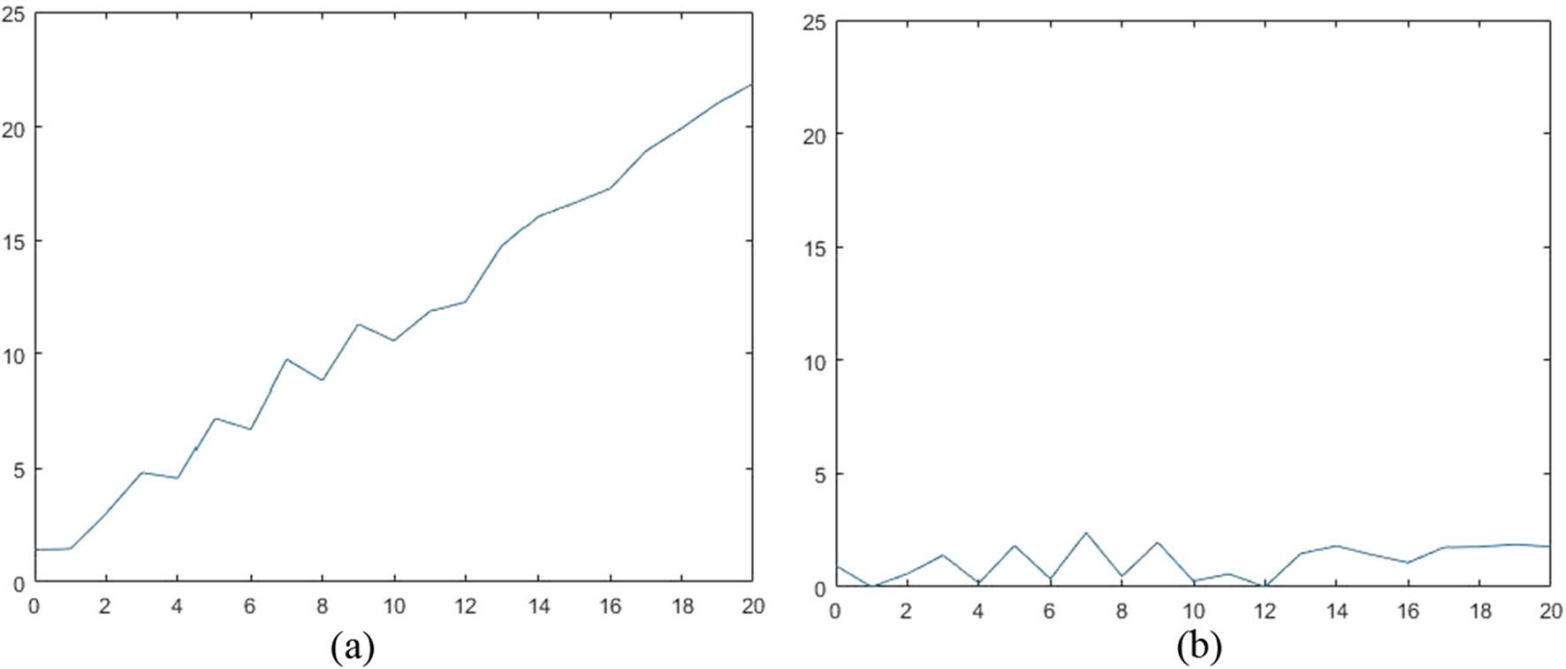
An NUC example based on simulated curve: (**a**) plot or simulated curve (**b**) plot of processed curve. The simulated curve is about a straight line with addictive white noise. After executing NUC, a similar noise figure is extracted for further study. Note that the algorithm is not used to remove line function but to highlight undulation.

An example based on a random curve (polylines made up by 21 points) is shown in Fig. 9 using (42) where the term ‘rand’ means a random value within interval [0,1]. As is seen, the fluctuation attached to rising trend is extracted and the resultant range is confined within local undulation indicated by random function. Note that the proposed method is not aimed at removing straight base line because the value of proceeded curve at every frequency sampling is no longer the random values employed in original **X**.

**Figure 10:**
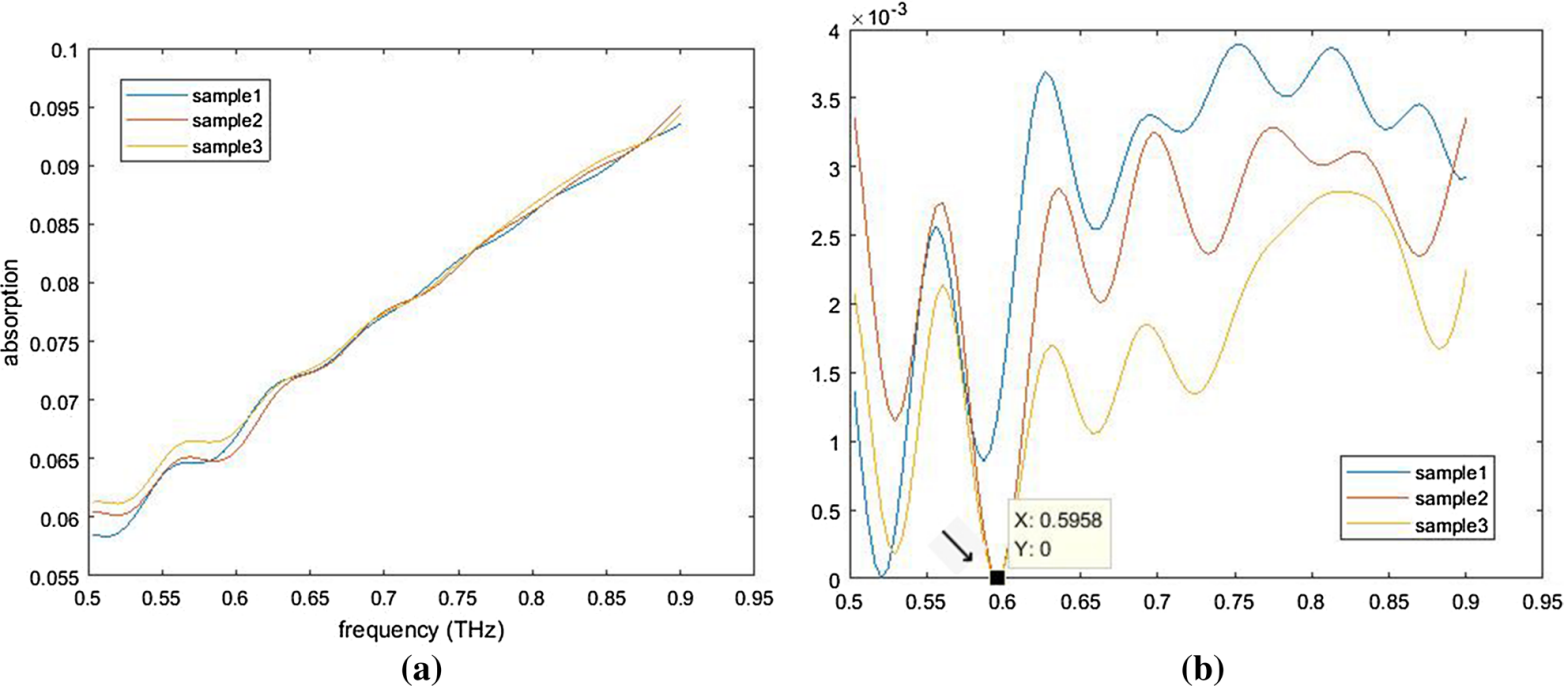
An NUC example based on extinction coefficient of colla corii asini: (**a**) plot of original curves (**b**) plot of processed curves. After excuting NUC, the undulation in Y-axis is reduced to only 1/9 of the original one. However, we do not assume the resultant curve as the standard spectrum. As is mentioned previously, the method is proposed for identification with CNN model at a low calculation cost.

Examples based on 3 real THz curves (extinction coefficient of colla corii asini*,* a well-known Chinese traditional medicine) are shown in Fig. 10 to show cases with more points. Given complex chemical constituents of individual samples and test errors, 3 samples have similar profiles but different details. As is seen, the range in Y axis of all curves are notably reduced after processing, and the difference between curves are magnified. Thus, more pixels can be employed to reflect detailed difference rather than supplementary area. Besides, the Y value of processed curve would be no less than 0 and the minimum of processed curve in Fig. 10b is marked by an arrow.

Although THz spectrum is predicted as a fingerprint spectrum, notable peaks are not observed for many cases, especially in tests on mixture as the spectra of various components overlap. As the example mentioned, colla corii asini is also named Ejiao in China, which is produced by boiling donkey hide as well as auxiliary materials and the chemical compound is very complex. A number of researchers focus on identify mixture without notable feature in THz band. NUC makes it possible to extract undulation for identification while removing overall linear trend that contributes to large range in absorption, therefore reducing pixels required for CNN model. An estimation can be made that original curve with 50 frequency samplings and absorption range of 0.045 need 50 × 450 = 22500 pixels according to Fig. 10a if range of 0.01 in absorption is quantified using 100 pixels. However, with the same ration of value to pixel, only 50 × 50 = 2500 pixels are needed to build a CNN model according to Fig. 10b.

42 $${\mathbf{X}} = \left[ {\begin{array}{*{20}c} {\begin{array}{*{20}c} 0 & {0 + {\text{rand}}} \\ \vdots & \vdots \\ \end{array} } \\ {\begin{array}{*{20}c} {19} & {19 + {\text{rand}}} \\ \end{array} } \\ {\begin{array}{*{20}c} {20} & {20 + {\text{rand}}} \\ \end{array} } \\ \end{array} } \right]$$

## Discussion

It is important to discuss the adaption of NUC before using it to pretreat THz curves. We start the discussion by analyzing necessary conditions for reasoning.

All of calculations are based on the convex hull of normalized curve. Therefore, the algorithm does not adapt to cases where convex hull does not exist (all points are in a line). This is seldom found in real practice because of random error and various values at multiple frequency. Thus, we assume the proposed method works for extracting undulation information if other condition is not considered.

In addition, the searching of point in convex hull is based on comparing slope and therefore it is important to assure that the slope can be calculated regardless of the shape of curve. All of points have different x coordinate before and after normalization. Consequently, the slope can’t approach infinity. Although the values at different frequency may equal, the slope can’t be 0 if two different points are involved in calculation (the cardinal number exceeds 1) because that the searching scope is beyond *a*_1_, *a*_2_, *b*_1_, *b*_2_. *P*_1_, *P*_2_, *Q*_1_, *Q*_2_ belong to A_3_, A_4_, B_3_, B_4_, respectively, only if the curve goes through (0, 1), (1, 1), (0, 0), (1, 0), respectively. In other words, set A_3_, A_4_, B_3_, B_4_ contain one or more non-zero elements or contain only one element 0. In order to calculate in all cases, we expand the definition of slope in narrow sense but define piecewise function ‘*slope*’ according to (31). It is expected that at least one of the *Ry*_*i*_ is minor than 1 because we want to reduce the undulation; the adaption is bad if Ry* ≥ 1. A detailed case study is listed in Table 1.

**Table 1:** Adaption analysis of algorithm: a case study by discussing numeric relationship between *a*_1_, *a*_2_, *b*_*1*_ and *b*_2_.

numeric relationship	*Ry*_1_ = 1 + (*b*_2_-*a*_1_)·|*k*_1_*|	*Ry*_2_ = 1 + (*a*_2_-*b*_1_)·|*k*_2_*|	*Ry*_3_ = 1 + (*a*_2_-*b*_1_)·|*k*_3_*|	*Ry*_4_ = 1 + (*b*_2_-*a*_1_)·|*k*_4_*|	adaption
*a*_1_ < *a*_2_ < *b*_1_ < *b*_2_	≥ 1	≤ 1	≤ 1	≥ 1	good
*a*_1_ < *b*_1_ < *a*_2_ < *b*_2_	≥ 1	≥ 1	≥ 1	≥ 1	bad
*a*_1_ < *b*_1_ < *b*_2_ < *a*_2_	≥ 1	≥ 1	≥ 1	≥ 1	bad
*b*_1_ < *b*_2_ < *a*_1_ < *a*_2_	≤ 1	≥ 1	≥ 1	≤ 1	good
*b*_1_ < *a*_1_ < *b*_2_ < *a*_2_	≥ 1	≥ 1	≥ 1	≥ 1	bad
*b*_1_ < *a*_1_ < *a*_2_ < *b*_2_	≥ 1	≥ 1	≥ 1	≥ 1	bad
*a*_1_ = *a*_2_ < *b*_1_ < *b*_2_	≥ 1	≤ 1	≤ 1	≥ 1	good
*b*_1_ < *a*_1_ = *a*_2_ < *b*_2_	≥ 1	≥ 1	≥ 1	≥ 1	bad
*b*_1_ < *b*_2_ < *a*_1_ = *a*_2_	≤ 1	≥ 1	≥ 1	≤ 1	good
*b*_1_ = *b*_2_ < *a*_1_ < *a*_2_	≤ 1	≥ 1	≥ 1	≤ 1	good
*a*_1_ < *b*_1_ = *b*_2_ < *a*_2_	≥ 1	≥ 1	≥ 1	≥ 1	bad
*a*_1_ < *a*_2_ < *b*_1_ = *b*_2_	≥ 1	≤ 1	≤ 1	≥ 1	good
*a*_1_ = *a*_2_ < *b*_1_ = *b*_2_	≥ 1	≤ 1	≤ 1	≥ 1	good
*b*_1_ = *b*_2_ < *a*_1_ = *a*_2_	≤ 1	≥ 1	≥ 1	≤ 1	good

As is found, if both *b*_2_ > *a*_1_, *a*_2_ > *b*_1_ are satisfied, the adaption is bad because *Ry*_i_ ≥ 1 for $\mathrm{i}=\in \{\mathrm{1,2},\mathrm{3,4}\}$. When the above mentioned two expressions are neither satisfied, we would conclude *b*_2_ < *a*_1_ ≤ *a*_2_ < *b*_1_ that conflicts with *b*_1_ ≤ *b*_2_. If *b*_2_ < *a*_1_ and *a*_2_ > *b*_1_, *k*_1_* ≠ 0 and *k*_4_* ≠ 0 because *a*_1_ ≠ 1 and *b*_2_ ≠ *N*. Thus, Ry* < 1; if *b*_2_ > *a*_1_ and *a*_2_ < *b*_1_, *k*_2_* ≠ 0 and *k*_3_* ≠ 0 because *a*_2_ ≠ *N* and *b*_2_ ≠ 1. In summarize, the algorithm adapts to process curves which are governed by (43). That’s the reason why a judgement is needed to check if the curve can be effectively processed by the algorithm. It turns out that after one shear transformation, the adjusted curve may be proceeded further as expression (43) is still met. Thus, one can iterate the process discussed above until expression (43) is no longer valid. The algorithm is destined to terminate after several circulations because every polygon has a dimension orthogonal to one of its edges, which is smaller than any other dimension.

43 $$\left( {b_{2} - a_{1} } \right)\left( {a_{2} - b_{1} } \right) < 0$$

The computation cost of algorithm is also concerned by potential users. The time required is tightly associate with shape of curve. Given number of points, the cost to obtain A, A_1_, A_2_ B, B_1_, B_2_ can be estimated. However, the cardinal number of A_3_, A_4_, B_3_, B_4_, would be greater for curves with drastic fluctuation than smooth curves, which may add pressure to slope calculation and comparison. Unlike other common methods to pretreat THz curve, including MSC, SG filter, median filter, the time cost varies notably.

The most suitable curves are estimated curves resemble that of colla corii asini, which have overall increasing or decreasing trend, large range but small local undulation relatively. After NUC, the range is compressed significantly, which allows fewer pixels to present supplementary space if quantification of absorption change over pixel is fixed. Considering that the scattering by grains tend to cause uptrend baseline in experiments conducted by THz-TDS, featureless smooth curve of some materials may magnify their local fluctuation after NUC in finer level, make it possible to identify them with CNN models.

## Conclusion

The conversion of THz spectroscopic data to 2-D image for CNN model can be achieved if a unit interval in absorption is quantified by definite number of pixels. Only a few pixels are hit by curve and the rest pixels do not provide effective information regarding curve shape. In order to reduce calculation cost of CNN, it is possible to reserve effective undulation and confine Y-range by adjusting curve in a normalized space and restore it into value-frequency space. Such operation is narrow undulation constraint (NUC), whose kernel thought is to confine curve with narrow parallel lines repeatedly and adjust range in Y by shear transformation.

A fast algorithm is proposed to achieve such goal whose kernel is searching critical points in the edge of convex hull and comparing slopes. The algorithm, described in several steps, is further illustrated and discussed from aspect of its adaption to THz curve. A number of set and definitions are purposefully built in this work, which facilitates understanding of calculations. The study suggests that studies on computer graphics also contributes to pretreatment of THz wave, which may be ignored in previous studies.

## Data Availability

The datasets generated during and/or analysed during the current study are available from the corresponding author on reasonable request.
